# ACTIONS TAKEN BY STROKE FAMILY CAREGIVERS TO POTENTIALLY PREVENT SURVIVORS’ HOSPITAL READMISSIONS IN CHINA

**DOI:** 10.1093/geroni/igz038.1062

**Published:** 2019-11-08

**Authors:** Hairui Yu, Jennifer Perion, Victoria Steiner, Linda Pierce

**Affiliations:** University of Toledo, Toledo, Ohio, United States

## Abstract

Stroke is a leading cause of death in China; its level of burden on the Chinese population is greater than the global average. Family caregiving plays an essential role in prevention and management of stroke. The purpose of this qualitative descriptive study was to identify actions family caregivers of stroke survivors in China take to prevent hospital readmissions. Using purposive sampling, adult family caregivers (n=10) were enrolled from Huai’an city in Jiangsu province who provided care for a survivor in community settings for six months or longer. Caregivers were asked questions in a face-to-face, semi-structured interview with content validity established by experts in the field. Audiotaped interviews were transcribed/translated into English and the narrative data analyzed using Colaizzi’s approach to content analysis. Seven female and three male caregivers with an average age of 55 years indicated actions that comprised six themes. These themes are: 1) Encouraging care recipients to be physically active, 2) Balancing a healthy diet with pleasurable foods, 3) Monitoring the physical health of care recipients and preventing injuries, 4) Developing personal and intimate strategies to motivate care recipients, 5) Providing emotional support and maintaining optimism, and 6) Gaining knowledge through relationships with doctors but desiring communication with other caregivers. Recognizing these actions taken by stroke family caregivers may improve education programs aimed at preventing hospital readmissions and be applicable throughout the world. Findings may also guide healthcare professionals who can advocate with Chinese local, provincial, and central health commissions on stroke survivors and their family caregivers’ behalf.

